# Genome-wide identification and expression analysis of the *CA* gene family in brown algae *Saccharina sculpera*

**DOI:** 10.3389/fpls.2026.1812032

**Published:** 2026-05-13

**Authors:** Xiaole Li, Xiaojie Li, Quansheng Zhang, Menghan Zhou, Lihao Guo, Junyi Wang, Mingyu Zhong

**Affiliations:** 1 School of Ocean, Yantai University, Yantai, China; 2 Shandong Oriental Ocean Sci-Tech Co., Ltd., Yantai, China

**Keywords:** bioinformatic analysis, carbonic anhydrase, expression analysis, gene family, *Saccharina sculpera*

## Abstract

**Introduction:**

Carbonic anhydrases (CAs) are zinc metalloenzymes that are ubiquitously distributed in nature and play important roles in the growth and development of diverse organisms. Although CAs have been extensively studied in plants and some algae, the *CA* gene family of *Saccharina sculpera*, an economically important seaweed, remains largely unexplored.

**Methods:**

In this study, the 18 identified *CA* genes were distributed on 7 chromosomes in the genome of *S. sculpera*, and classified into α-, β-, and γ-subfamilies.

**Results:**

Physicochemical properties and subcellular localization analysis revealed that these CAs were hydrophilic, acidic and unstable proteins, primarily localized to chloroplasts and mitochondria. Gene structure analysis demonstrated that *CA* genes share similar characteristics within the same subfamily, while collinearity analysis revealed that tandem duplication is the primary mechanism driving *CA* gene family expansion. Additionally, a close phylogenetic relationship was found between the *CA* genes of *S. sculpera* and *Saccharina japonica*. Promoter cis-element analysis indicated that light is the dominant regulatory factor. Furthermore, RT-qPCR revealed distinct temporal variation in *CA* gene expressions, with most genes (*SscCA2/4/6/7/11-18*) decreasing initially then rising and the remaining (*SscCA3/5/8/9*) declining continuously throughout the developmental stages. In addition, enzyme activity analysis showed that total CA activity gradually increased throughout the developmental stages.

**Discussion:**

This systematic study of the *CA* gene family provides a foundation for further investigations into the biological functions of *CA* genes in *S. sculpera*.

## 1 Introduction

*Saccharina sculpera*, previously known as *Kjellmaniella crassifolia*, is a brown alga widely distributed in the southern regions of Hokkaido and the northern areas of the east coast of the Korean Peninsula (Tang et al., 2022). Notably, *S. sculpera* is abundant in fucoidan, a polysaccharide with a broad range of biological activities, including immunopotentiation (Berteau, 2003), anti-cancer (Han et al., 2014), antitumor (Zaporozhets et al., 2014) and blood glucose regulation effects (Tang et al., 2025). These properties make fucoidan a promising candidate for the development of high value-added health supplements and pharmaceuticals. Thus, *S. sculpera*, as a high production species of fucoidan, possesses significant economic value. Furthermore, studies have demonstrated that brown algae have a higher carbon sequestration capacity than most red algae and green algae, and play a vital role as important carbon sinks in the marine environment (Yan-Hui et al., 2019).

Carbonic anhydrases (CAs) are metalloenzymes that predominantly contain zinc ions, with a very small number of cobalt or cadmium ions as the metal complex. These enzymes catalyze the reversible hydration reaction of carbon dioxide (CO_2_), rapidly converting CO_2_ to bicarbonate (HCO_3_^−^) and vice versa. To date, based on amino acid sequence similarity, the *CA* gene family is currently divided into eight evolutionarily distinct subtypes, including α-CA, β-CA, γ-CA, δ-CA, η-CA, ζ-CA, θ-CA and ι-CA. All CA subtypes are found in algae, except for η-CA, which is only found in *Plasmodium falciparum* (DiMario et al., 2018; Nocentini et al., 2021). For example, 19 genes encoding CA subtypes have been identified in the *Arabidopsis thaliana* genome, including 8 α-CA, 6 β-CA and 5 γ-CA (Feng et al., 2020). Similarly, in *Chlamydomonas reinhardtii*, 15 CA subtypes have been identified, comprising 3 α-CA, 9 β-CA and 3 γ-CA (Aspatwar et al., 2018). In *Saccharina japonica*, 11 *CA* genes have been detected, with 5 belonging to α-CA, 3 to β-CA and 3 to γ-CA (Wang et al., 2024b). Additionally, *Phaeodactylum tricornutum* possesses 9 CA subtypes, including 5 α-CA, 2 β-CA and 2 γ-CA (Tachibana et al., 2011). Meanwhile, 13 CA genes are present in *Thalassiosira pseudonana*, consisting of 3 α-CA, 5 γ-CA, 4 δ-CA and 1 ζ-CA (Tachibana et al., 2011). In plants and algae, CAs are mainly distributed in subcellular compartments such as chloroplasts, mitochondria, and cytoplasm, where they exert different physiological functions (Ignatova et al., 2019). In *C. reinhardtii*, mitochondrial β-CA isoforms (CAH4/5) supply CO_2_ or HCO_3_^−^ for specific metabolic reactions, including the carboxylation reaction catalyzed by Rubisco, which is the first step of fatty acid biosynthesis mediated by acetyl-CoA carboxylase, and reversible reactions within the mitochondria (Moroney et al., 2011). On the other hand, α-CA proteins (CAH1/2), located in the periplasmic space, deliver inorganic carbon to the appropriate intracellular sites. Meanwhile, CAs play vital roles in preventing CO_2_ leakage from the cell and maintaining the optimal intracellular pH range (Moroney et al., 2011). Further, in higher plants, γ-CA associates with the mitochondrial NADH dehydrogenase complex (Complex I) (Maldonado et al., 2020).

So far, systematic investigations have been conducted on CAs in model species of cyanobacteria, green algae and diatoms, confirming that they play an important role in the carbon-concentrating mechanism (CCM) (Price et al., 1998; Giordano et al., 2005; Jensen et al., 2020; Burlacot and Peltier, 2023). In brown algae, the research has primarily focused on *S. japonica*, where the transcript levels of α-CA (Sjα-CA2) were localized in the periplasmic space of its gametophytes and are induced in response to high concentrations of HCO_3_^−^ (Bi et al., 2021). Additionally, β-CA has been identified as a key enzyme in the CCM, where it mediates the conversion of over 85% of HCO_3_^−^ in seawater into CO_2_ for photosynthesis. Furthermore, β-CA genes are highly expressed in the young sporophyte stage, and their transcript abundance is positively correlated with environmental HCO_3_^−^ concentration (Hao et al., 2023). Nevertheless, the transcription level of mitochondrial γ-CA is only induced by low CO_2_ concentrations (Bi et al., 2021a). In contrast, previous studies on *S. sculpera* have primarily addressed the application of fucoidan and the influence of environmental factors such as light and temperature on its growth (Qu et al., 2019; Ren et al., 2019). To date, the *CA* gene family has not been investigated in *S. sculpera*. In this study, we performed a comprehensive genome-wide bioinformatic analysis of the *CA* gene family in *S. sculpera*, including the identification of their members, phylogeny, gene structures, motif compositions, conserved domains, chromosomal locations, promoter cis-elements, and gene duplication events. Besides, synteny analyses of the *CA* gene members among *S. sculpera*, *Cladosiphon okamuranus* and *Ectocarpus siliculosus* were performed. *S. sculpera* samples for analysis of CA expressions and enzyme activities were collected from four periods (see details in section 2.6). These periods basically cover the main growth and development stages of the seaweed, and were roughly equivalent to the four crucial stages (uneven, tender-thin, mature thick and maturation stages) of *S. japonica* (a close relative of *S. sculpera*) growth and development (Wang et al., 2020). Therefore, analyzing samples selected in these periods will probably provide a relatively comprehensive and representative atlas of CA gene expressions and CA activities in *S. sculpera*. In summary, this study provides a valuable reference for future functional investigations of *CA* genes in *S. sculpera*. The bioinformatics analyses clarify carbon utilization in marine algae and contribute to the scientific understanding required to enhance marine carbon cycling and mitigate global warming.

## 2 Materials and methods

### 2.1 Identification and phylogenetic analysis

Sequences of *CA* genes from *A. thaliana* and *E. siliculosus* were downloaded from The Arabidopsis Information Resource database (TAIR, https://www.arabidopsis.org/results?mainType=general&searchText=Carbonic%20Anhydrase&category=genes) and the NCBI database (https://www.ncbi.nlm.nih.gov/; GenBank accession: GCA_000310025.1). These sequences were used as queries in BLASTP searches against the *S. sculpera* genome (Guo et al., 2025),with an E value was set to ≤ 1×10–^5^ for screening candidate sequences. The HMM profiles of the *Carb_anhydrase* (PF00194), *Pro_CA* (PF00484), and *Hexapep* (PF00132) domains were obtained from the Pfam database (http://pfam.xfam.org/) (Wang et al., 2023) while HMMER v3.0 software was run with default parameters to identify CA sequences in *S. sculpera* (Finn et al., 2011). The results from BLASTP and HMMER were combined and deduplicated. All the putative CA sequences were subsequently validated on InterPro (https://www.ebi.ac.uk/interpro/) and NCBI Batch-CD-Search (https://www.ncbi.nlm.nih.gov/Structure/bwrpsb/bwrpsb.cgi) as described previously (Marchler-Bauer et al., 2015).

The CA protein sequences of *S. japonica* (Li et al., 2025), *C. reinhardtii* (Moroney et al., 2011), *P. tricornutum* (Bowler et al., 2008; Tachibana et al., 2011) and *Gracilariopsis chorda* (Lee et al., 2018) were obtained from the published sources. Multiple sequence alignments were performed between *S. sculpera* CA proteins and those from *A. thaliana*, *E. siliculosus*, *S. japonica*, *C. reinhardtii*, *P. tricornutum* and *G. chorda*, using the MUSCLE program. A phylogenetic tree of *CA* gene families in *S. sculpera* and these other species was constructed using the neighbor-joining method of the MEGAX64 software, with 1000 bootstrap replications. The resulting tree was further visualized and edited using iTOL v7 (https://itol.embl.de/) (Letunic and Bork, 2024).

### 2.2 Physicochemical properties and subcellular localization analysis

The ExPASy ProtParam tool (https://web.expasy.org/protparam/) was used to analyze the molecular weight, isoelectric point (pI), hydrophilicity, hydrophobicity and stability of CA proteins (Artimo et al., 2012). Subcellular localization of the CA proteins was predicted using WoLF PSORT (https://wolfpsort.hgc.jp/) (Huang et al., 2021).

### 2.3 Gene structure, conserved motifs and conserved domains analysis

The exons and introns of the *CA* gene family were obtained from the annotation file (unpublished data) of the *S. sculpera* genome for the gene structure analysis. The conserved motifs were predicted using the online tool MEME (http://meme-suite.org/tools/meme) (Bailey et al., 2009), with the maximum number of motifs set to 20 and all other parameters set to default values. Conserved domains of identified CA proteins were analyzed using NCBI Batch-CD-Search. The distribution of *CA* gene structure, conserved motifs and conserved domains was visualized using the gene structure view function in TBtools (Chen et al., 2020).

### 2.4 Chromosomal distribution, gene duplication and synteny analysis

According to the annotation information of the *S. sculpera* genome, the chromosome location information of *CA* gene family was obtained. Gene duplication events within the CA genes were analyzed using the Multiple Collinearity Scan Toolkit X (MCScanX-master) on the Linux operating system. TBtools was used to map the chromosomal locations of CA genes, mark tandem duplicated genes and calculate the Ka/Ks ratios.

Further investigate the evolution process of the *CA* gene family, the representative species, including *C. okamuranus*, *E. siliculosus, S. japonica*, *C. reinhardtii*, *G. chorda* and *A. thaliana*, were selected for comparative genomics. The respective genome sequences were compared with those of *S. sculpera* to identify collinear relationships between *S. sculpera* and other species. TBtools was then used to generate partial collinearity plots between *S. sculpera* and each of the selected species.

### 2.5 Promoter cis-elements analysis

The sequences of 2000 bp from the promoter region of the CA genes were extracted using TBtools. Followed by cis-acting regulatory element prediction in these sequences using PlantCare (http://bioinformatics.psb.ugent.be/webtools/plantcare/html/) (Rombauts et al., 1999). The predicted elements were subsequently visualized using TBtools.

### 2.6 Quantitative reverse transcriptional PCR

Samples were collected in December 23, 2022; January 13, 2023; March 5, 2023 and April 21, 2023 from the coastal waters of Dongchu Island, Weihai, Shandong Province, China. After collection, the sample surfaces were quickly rinsed with sterile distilled water, then immediately frozen and stored in liquid nitrogen. Total RNA was extracted from samples at different developmental stages using SPARKeasy Plant RNA Kit (SparkJade, China), following the manufacturer’s instructions. RNA quality was assessed by agarose gel electrophoresis and NanoQuant (TECAN, Switzerland). Subsequently, 1 μg of the total RNA was reverse transcribed into cDNA using the HiScript^®^ III RT SuperMix for qPCR (+gDNA wiper) (Vazyme, Nanjing, China) following the manufacturer’s instructions. The RT-qPCR assays were conducted using a Bio-Rad CFX96 Real-Time PCR System using ChamQ Blue Universal SYBR qPCR Master Mix (Vazyme). The primers used in RT-qPCR are provided in **Table 1**. The gene Sscptg001662lG000020.1 from *S. sculpera*, which was renamed NC3, was used as the housekeeping gene. The RT-qPCR program was set as follows: 94 °C for 3 min, followed by 40 cycles of 94 °C for 30 s, 58 °C for 30s and 72 °C for 1 min. Each reaction was performed in three biological replicates. The relative expression level of each gene was calculated using the 2^–ΔΔCT^ method (Ma et al., 2021).

**Table 1:** Primer sequences of *CA* genes.

Primer name	Primer sequences
NC3-F	5’ GGTACGCTCTGCCCCAC 3’
NC3-R	5’ GCGGTCTTCATCTCCTGGT 3’
SscCA1-F	5’ TGCAGCGAGATAGTGAGGT 3’
SscCA1-R	5’ GGGCGGTTTGTGTTTGA 3’
SscCA2-F	5’ TGGGAGGTGCTGGGTTC 3’
SscCA2-R	5’ CAGGGAGGGGTGGTGAG 3’
SscCA3-F	5’ TGATCGGCGACACACAG 3’
SscCA3-R	5’ TCAAACCACGAAAACGG 3’
SscCA4-F	5’ CGTACGAGCTGATACCGTCC 3’
SscCA4-R	5’ CTCACTATCTCGCTGCAGGG 3’
SscCA5-F	5’ GCACGGAGGGAGTGAAG 3’
SscCA5-R	5’ TGTGGAAGACGGCAATG 3’
SscCA6-F	5’ TGGTAGCCGTTTTTCTGG 3’
SscCA6-R	5’ GTGTCGGTCGTCGTTGTC 3’
SscCA7-F	5’ CGTGGAGACGGTGGTGG 3’
SscCA7-R	5’ TCGTTCGCTTGGAAAAATAG 3’
SscCA8-F	5’ ACCACCCGCATCTACGA 3’
SscCA8-R	5’ TCCACTTCACGCCCTCA 3’
SscCA9-F	5’ CGGTGTTCTCGCACTACA 3’
SscCA9-R	5’ ATCCATTTTACGCCCTCT 3’
SscCA10-F	5’ CTCCCTCCGAGCACATGATC 3’
SscCA10-R	5’ AAGTGTCGAACATCACGCCT 3’
SscCA11-F	5’ AGCGACCCCGTCACCAT 3’
SscCA11-R	5’ TACCCCGTTCATCCACCTT 3’
SscCA12-F	5’ CCAGGGAGAGGACATGGAGA 3’
SscCA12-R	5’ CGACAAGTTGATGGGGCTCT 3’
SscCA13-F	5’ ACTCGCACTACATGGGGTCT 3’
SscCA13-R	5’ AATCGTGGGGTCGGTCA 3’
SscCA14-F	5’ GTTCCTCTTCATTGGGTGCT 3’
SscCA14-R	5’ CGATGATGTGCTTCACCTTC 3’
SscCA15-F	5' AGCTGAACGTGCAGGAGT 3'
SscCA15-R	5' ACAAGGCGAACGAAGAAT 3'
SscCA16-F	5’ GGCTGTCATTCGAGGCGATA 3’
SscCA16-R	5’ CGATGATGACCCAGGAGTCG 3’
SscCA17-F	5’ GGGAACAAGCCCACCATCG 3’
SscCA17-R	5’ CCTTCGCCCCGTCCATCA 3’
SscCA18-F	5’ GCGATGGTCGGCATGAAATC 3’
SscCA18-R	5’ GTTTCCTCCCCATACCTGCC 3’

### 2.7 Carbonic anhydrase activity measurement

Enzyme activity assays were performed using samples collected during the same periods as the RT qPCR analysis. The Carbonic Anhydrase (CA) Activity Assay Kit (Mlbio, China) was used to determine the enzyme activity, following the manufacturer’s instructions. The enzyme activity unit was defined as the amount of enzyme that catalyzes the formation of 1 μmol p-nitrophenol per minute per gram of frozen tissue at room temperature. Each experiment group was repeated three replicates.

### 2.8 Statistical analyses

The results of RT−qPCR and CAs activity were visualized using GraphPad Prism 10.1.2. Statistical analyses were performed using SPSS Statistics 26 software. The homogeneity of variance test was conducted on the data, and one-way analysis of variance (One-way ANOVA) was adopted to compare differences among groups, with the significance level set at 0.05. All the error bars were standard deviation (SD) from the independent biological replicates.

## 3 Results

### 3.1 Identification and phylogenetic analysis

**Figure 1:**
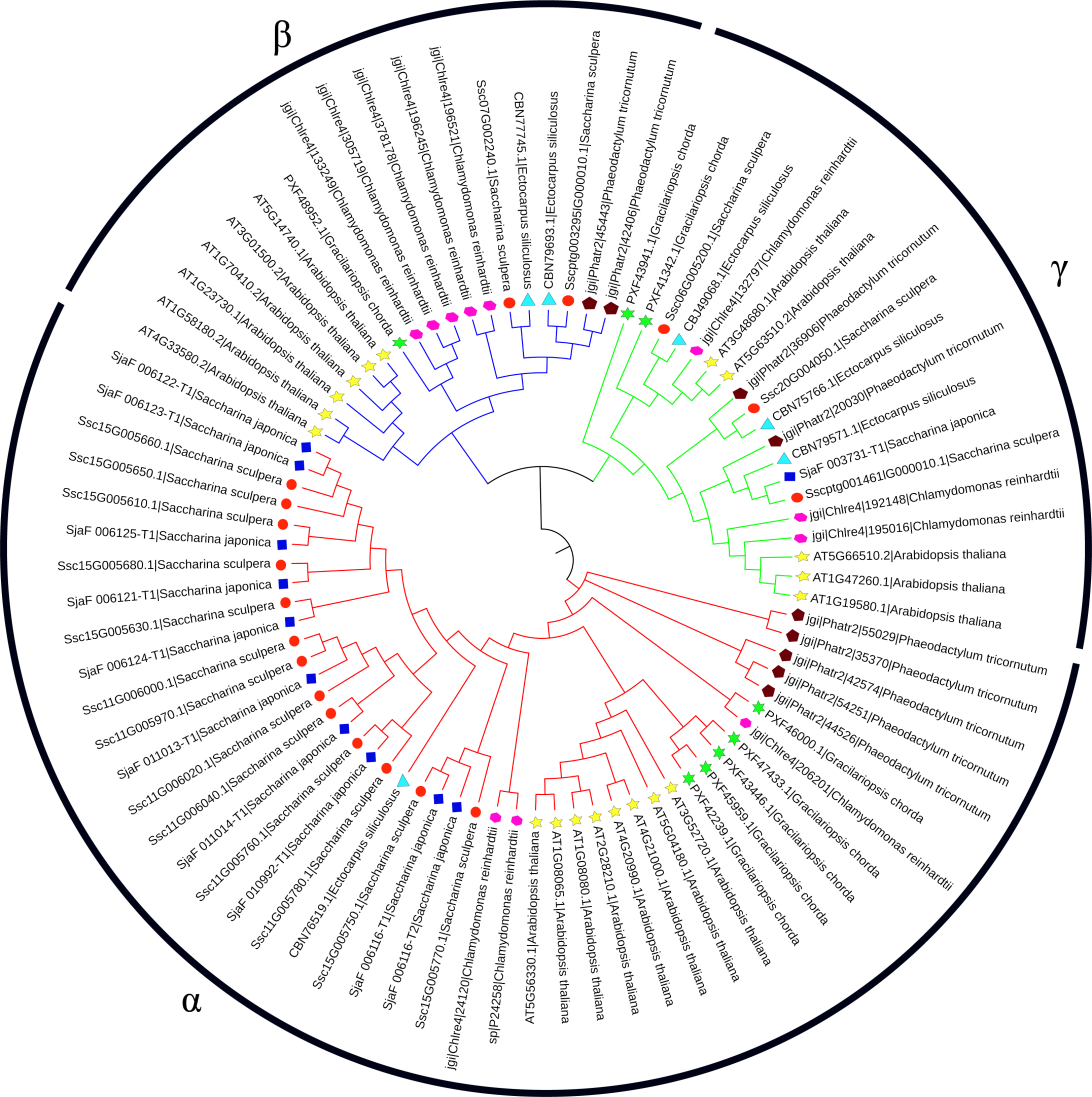
Phylogenetic tree of CA proteins in algae and *A. thaliana*. Different colors represent different species. *S. sculpera* is marked with red circles, *S. japonica* with dark blue squares, *A. thaliana* with yellow five-pointed stars, *E. siliculosus* with light blue triangles, *C. reinhardtii* with pink hexagons, *G. chorda* with green hexagons, and *P. tricornutum* with brown pentagons. Branches in the phylogenetic tree colored red, blue, and green represent the α-CA, β-CA, and γ-CA subfamilies, respectively.

A total of 18 CAs were identified in the genome of *S. sculpera*. To analyze the evolutionary relationships of these CAs, a phylogenetic tree was constructed using the identified CA sequences (**Figure 1**). The results showed that CAs from different species within the same subfamily clustered together, with the CAs of *S. sculpera* belonging to three subfamilies, including α-CA, β-CA and γ-CA. The α-CA subfamily was the largest subfamily containing 13 members (*SscCA1* to *SscCA13*), while the β-CA and γ-CA subfamilies contained 2 members (*SscCA14* and *SscCA15*) and 3 members (*SscCA16* to *SscCA18*), respectively.

### 3.2 Physicochemical properties and subcellular localization analysis

The physicochemical properties of the identified CAs are summarized in **Table 2**. The results showed that their amino acid (aa) sequence lengths and protein molecular weights exhibited significant differences, ranging from 202 aa (*SscCA16*) to 557 aa (*SscCA15*) and from 20.83 kDa (*SscCA16*) to 61.61 kDa (*SscCA15*), respectively. The CAs also displayed substantial variations in isoelectric point (pI) and instability index. The pI values ranged from 4.37 (*SscCA9*) to 9.04 (*SscCA16*). Overall, 15 members were classified as acidic with pI < 7, while 3 members were basic with pI > 7. The instability index varied from 21.13 (*SscCA18*) to 60.96 (*SscCA1* and *SscCA2*), with 83% of the members having an instability index greater than 40. The aliphatic index ranged from 69.01 (*SscCA14*) to 82.1 (*SscCA13*), where a higher value indicates higher thermal stability of the CA proteins. Regarding the grand average of hydropathicity (GRAVY), all CAs had GRAVY ranging from -0.527 (*SscCA14*) to 0.234 (*SscCA16*). Subcellular localization predictions (**Table 2**) indicated that the CAs are broadly distributed across various cellular compartments. Specifically, *SscCA1*, *SscCA2*, *SscCA3*, *SscCA5* and *SscCA18* were localized to the mitochondria. *SscCA4*, *SscCA6*, *SscCA14*, *SscCA15* and *SscCA16* to the chloroplasts, and *SscCA8*, *SscCA10* and *SscCA11* to the nucleus. Additionally, *SscCA12* and *SscCA13* were found in the cell wall, *SscCA7* in the vacuole, *SscCA9* in the cytoplasm, and *SscCA17* in the peroxisome.

**Table 2:** Information of 18 *CA* genes in *S. sculpera*.

Sequence ID	Gene name	Number of amino acid	Molecular weight (kDa)	Theoretical pI	Instability index	Aliphatic index	Grand average of hydropathicity	Subcellular location
Ssc11G006000.1	*SscCA1*	295	32.16	4.61	60.96	76.71	-0.296	mitochondrion
Ssc11G005970.1	*SscCA2*	295	32.16	4.61	60.96	76.71	-0.296	mitochondrion
Ssc11G006020.1	*SscCA3*	295	31.95	4.4	54.77	80.98	-0.178	mitochondrion
Ssc11G006040.1	*SscCA4*	292	31.67	5.48	49.77	78.49	-0.284	chloroplast
Ssc11G005780.1	*SscCA5*	307	34.17	4.81	57.61	76.78	-0.354	mitochondrion
Ssc11G005760.1	*SscCA6*	293	32.34	4.82	59.59	80.14	-0.258	chloroplast
Ssc15G005630.1	*SscCA7*	294	32.77	4.38	50.97	76.22	-0.254	vacuole
Ssc15G005680.1	*SscCA8*	279	31.50	5.11	46.55	69.5	-0.495	nucleus
Ssc15G005610.1	*SscCA9*	245	27.69	4.37	49.37	74.29	-0.249	cytoplasm
Ssc15G005660.1	*SscCA10*	236	26.80	4.5	53.92	76.78	-0.297	nucleus
Ssc15G005650.1	*SscCA11*	272	30.68	4.54	49.96	69.82	-0.432	nucleus
Ssc15G005750.1	*SscCA12*	290	32.26	4.43	52.28	73.24	-0.363	cell wall
Ssc15G005770.1	*SscCA13*	229	25.09	4.46	50.91	82.1	-0.121	cell wall
Sscptg003295lG000010.1	*SscCA14*	424	45.85	8.81	43.67	69.01	-0.527	chloroplast
Ssc07G002240.1	*SscCA15*	557	61.61	7.05	40.9	79.84	-0.29	chloroplast
Ssc06G005200.1	*SscCA16*	202	20.83	9.04	34.53	91.19	0.234	chloroplast
Sscptg001461lG000010.1	*SscCA17*	304	31.26	4.9	31.41	80.86	-0.071	peroxisome
Ssc20G004050.1	*SscCA18*	209	20.75	5.44	21.13	89.19	0.278	mitochondrion

### 3.3 Gene structure, conserved motifs and conserved domains analysis

**Figure 2:**
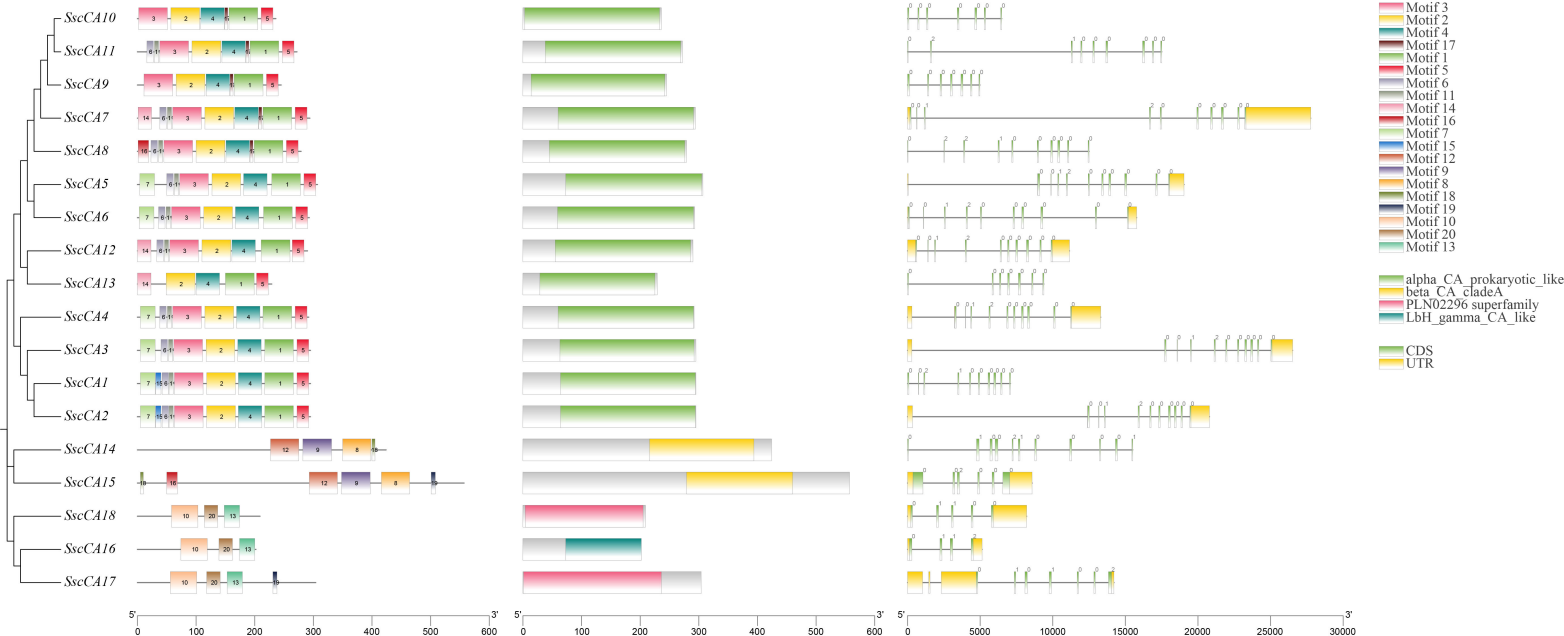
Analysis of conserved motifs, conserved domains and gene structures of CAs.

Distinct differences exist in both the position and number of conserved motifs across the three CA subfamilies (**Figure 2**), with all members of the α-CA subfamily containing Motif 1, Motif 2, Motif 3, Motif 4 and Motif 5. The β-CA subfamily uniformly contained Motif 8, Motif 9 and Motif 12, while the γ-CA subfamily consistently possessed Motif 10, Motif 13 and Motif 20. Conserved protein domain analysis revealed that members within each subfamily shared the characteristic protein domains or superfamilies unique to that subfamily. Further analysis of the gene structure of the CA family members showed that, although members of the same subfamily do not display overtly similar gene structures, they exhibit comparable exon numbers, generally ranging from 4 to 11, with the majority possessing approximately 7 exons. For example, the 13 α−CA subfamily members had either 7 or 10 exons. Of the 2 members in the β−CA subfamily, *SscCA14* contained 11 exons, whereas *SscCA15* had 6, and in the 3 γ-CA subfamily members, the number of exons ranges from 4 to 7.

### 3.4 Chromosomal distribution, gene duplication and synteny analysis

**Figure 3:**
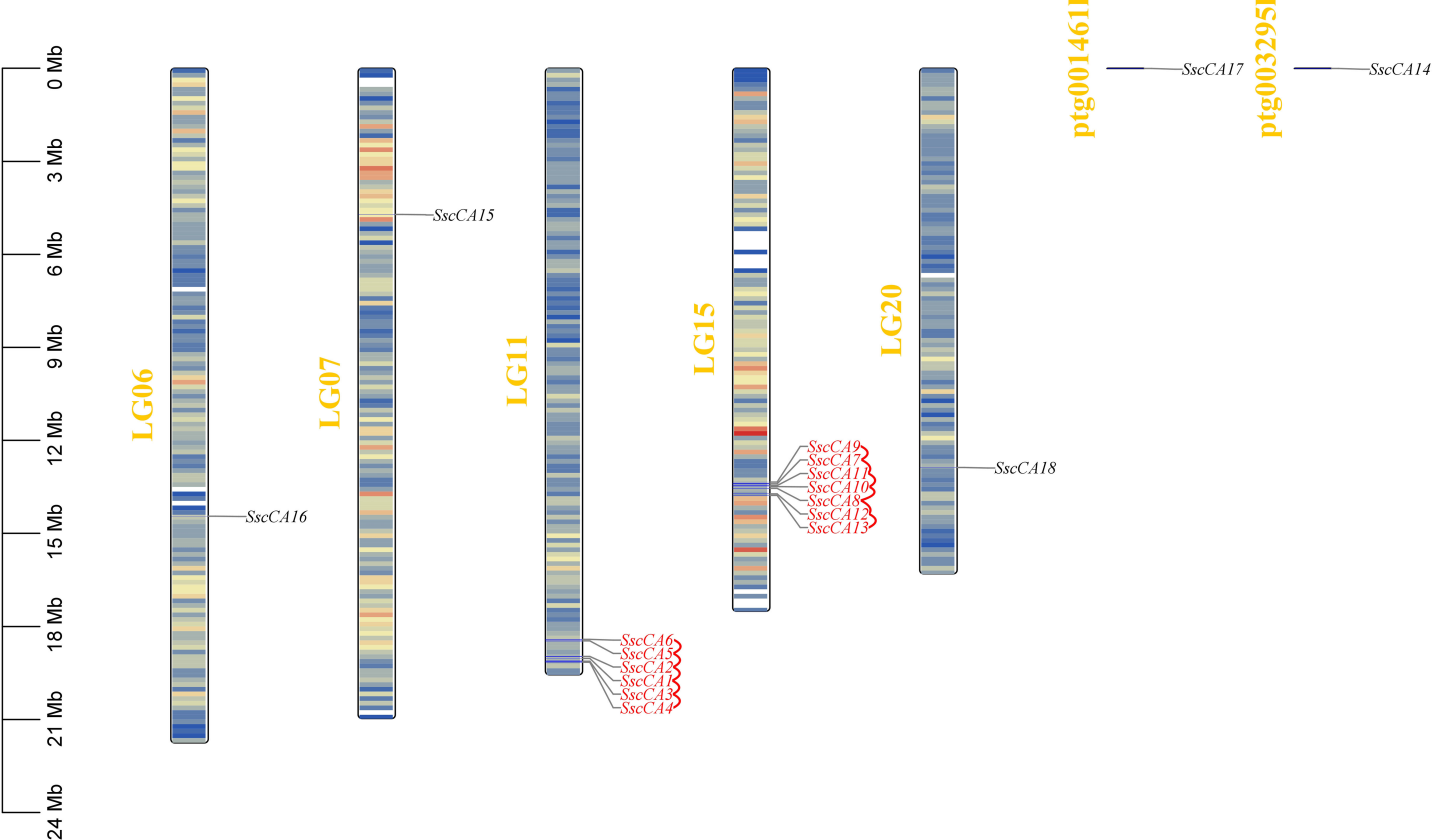
Chromosomal localization of *CA* genes. Genes marked by red lines indicate tandem duplication events.

Chromosomal mapping showed that CA genes were unevenly distributed across the 7 chromosomes of *S. sculpera* (**Figure 3**). Specifically, 1 CA member was localized to each of chromosomes LG06, LG07, LG20 and the scaffold chromosomes ptg001461l and ptg003295l, while 6 members were mapped to chromosomes LG11, and 7 on chromosome LG15. Further analysis of the *CA* gene family duplication patterns identified 13 tandemly duplicated genes, representing approximately 72% of all CA family members. These genes were exclusively present on chromosomes LG11 and LG15, and belonged to the α−CA subfamily. According to Ka/Ks calculations (**Table 3**), both the Ka and Ks values for *SscCA1*/*SscCA2* and *SscCA12*/*SscCA13* were 0, whereas the Ka/Ks ratios for all other tandemly duplicated gene pairs were less than 1.

**Table 3:** Ka/Ks ratios of each pair of tandem duplicated *CA* genes.

Duplicated gene pairs	Ka	Ks	Ka/Ks
*SscCA5*/*SscCA6*	0.15	0.49	0.31
*SscCA2*/*SscCA5*	0.33	0.71	0.47
*SscCA1*/*SscCA2*	0.00	0.00	NaN
*SscCA1*/*SscCA3*	0.11	0.33	0.33
*SscCA3*/*SscCA4*	0.13	0.32	0.42
*SscCA7*/*SscCA9*	0.11	0.38	0.30
*SscCA7*/*SscCA11*	0.11	0.38	0.30
*SscCA10*/*SscCA11*	0.05	0.41	0.12
*SscCA8*/*SscCA10*	0.12	0.42	0.28
*SscCA8*/*SscCA12*	0.53	1.00	0.53
*SscCA12*/*SscCA13*	0.00	0.00	NaN

**Figure 4:**
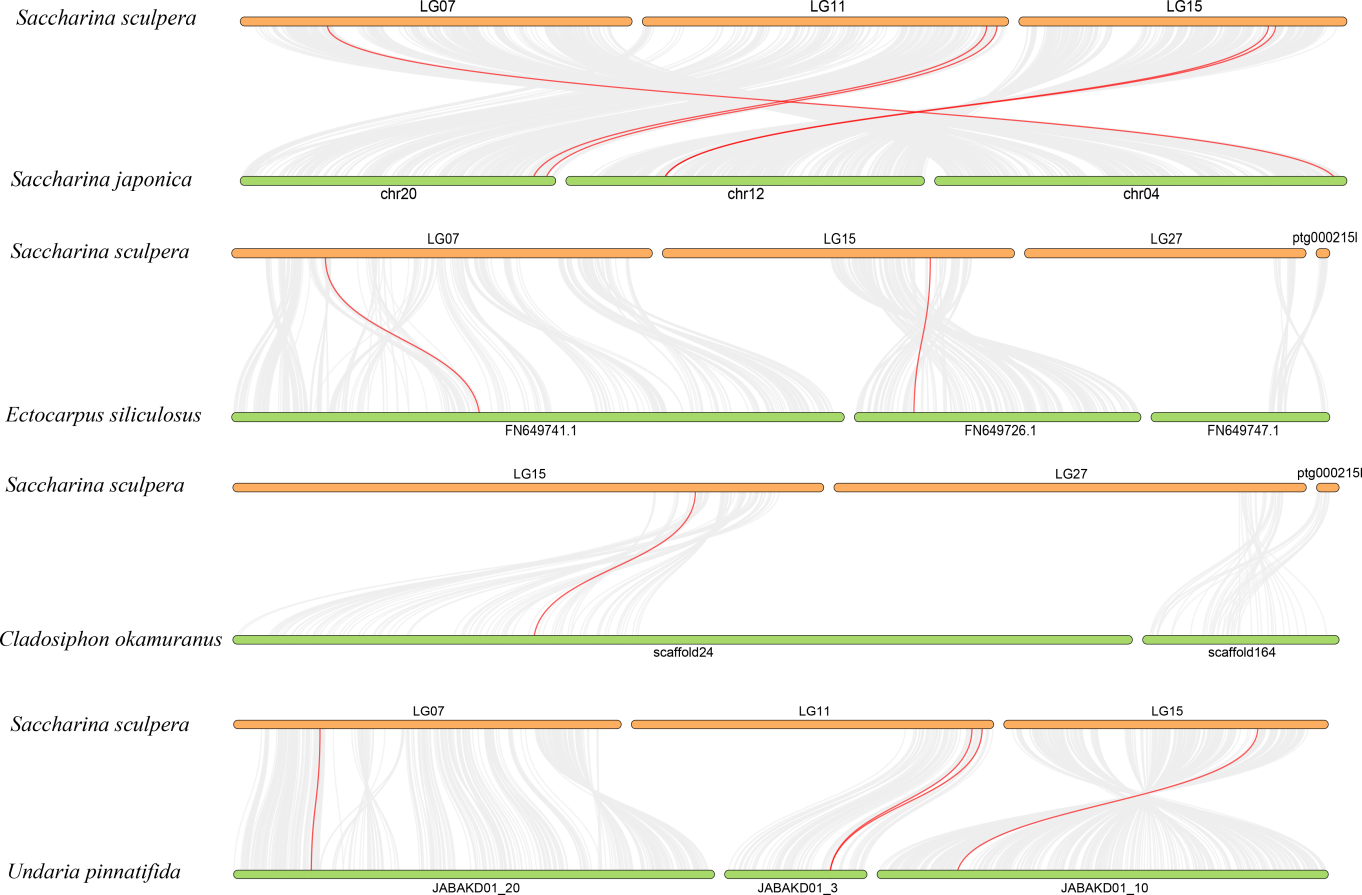
Synteny analysis of *CA* genes between *S. sculpera*, *S. japonica*, *E. siliculosus*, *C. okamuranus*, and *U. pinnatifida*. The syntenic CA gene pairs between *S. sculpera* and other species were highlighted with the red lines.

Collinearity analysis was performed between the *CA* genes of *S. sculpera* in comparison with those from *C. okamuranus*, *E. siliculosus*, *S. japonica*, *Undaria pinnatifida*, *C. reinhardtii*, *G. chorda* and *A. thaliana*. The results revealed that *S. sculpera* shared 5 collinear gene pairs with *S. japonica*, 2 with *E. siliculosus*, 1 with *C. okamuranus* and 4 with *U. pinnatifida*. Nevertheless, no collinear gene pairs were detected between *S. sculpera* and *C. reinhardtii*, *G. chorda* or *A. thaliana* (**Figure 4**).

### 3.5 Promoter cis-elements analysis

**Figure 5:**
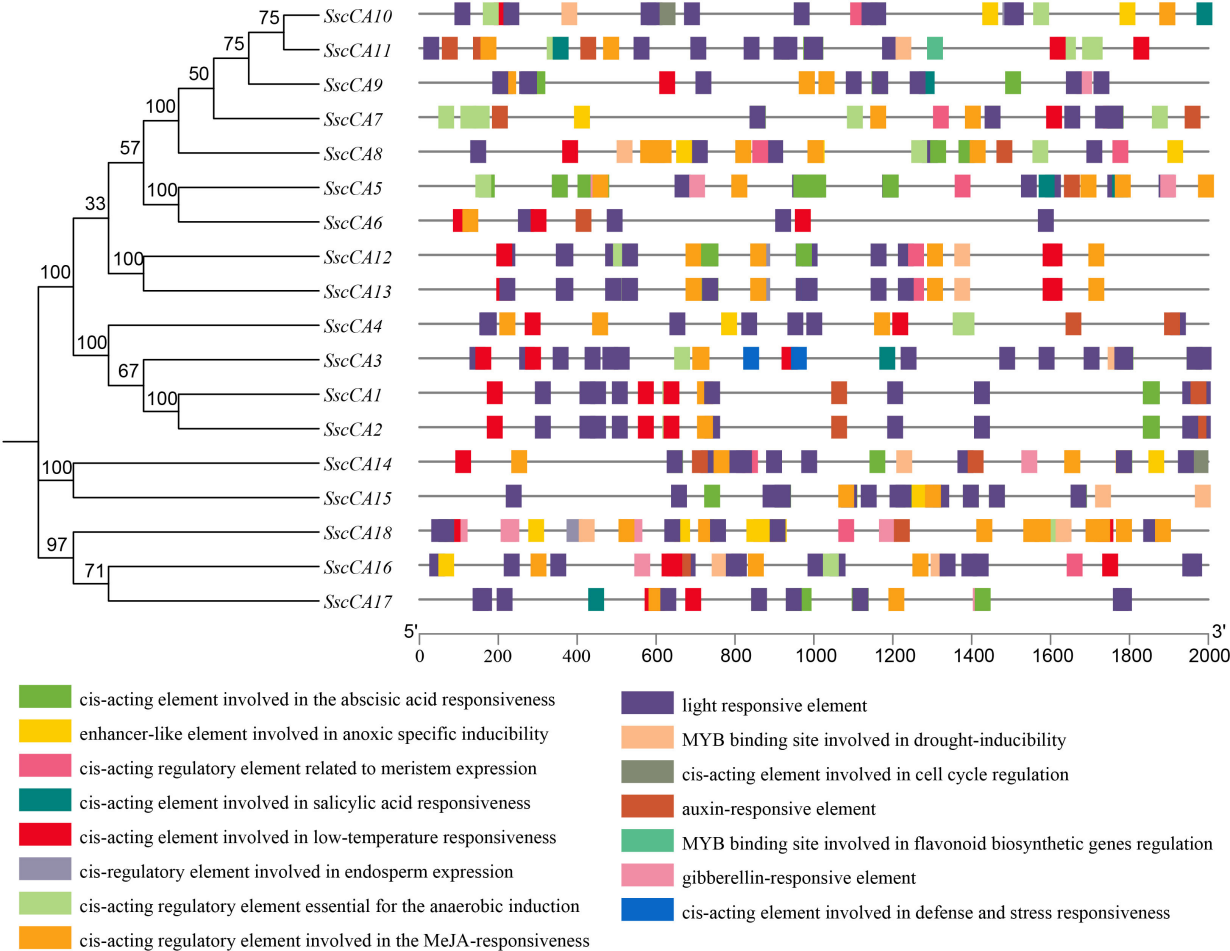
Analysis of promoter cis-acting elements of *CA* genes. Boxes of different colors represent distinct types of cis-acting elements, and gray lines indicate the lengths of the gene promoters.

**Figure 6:**
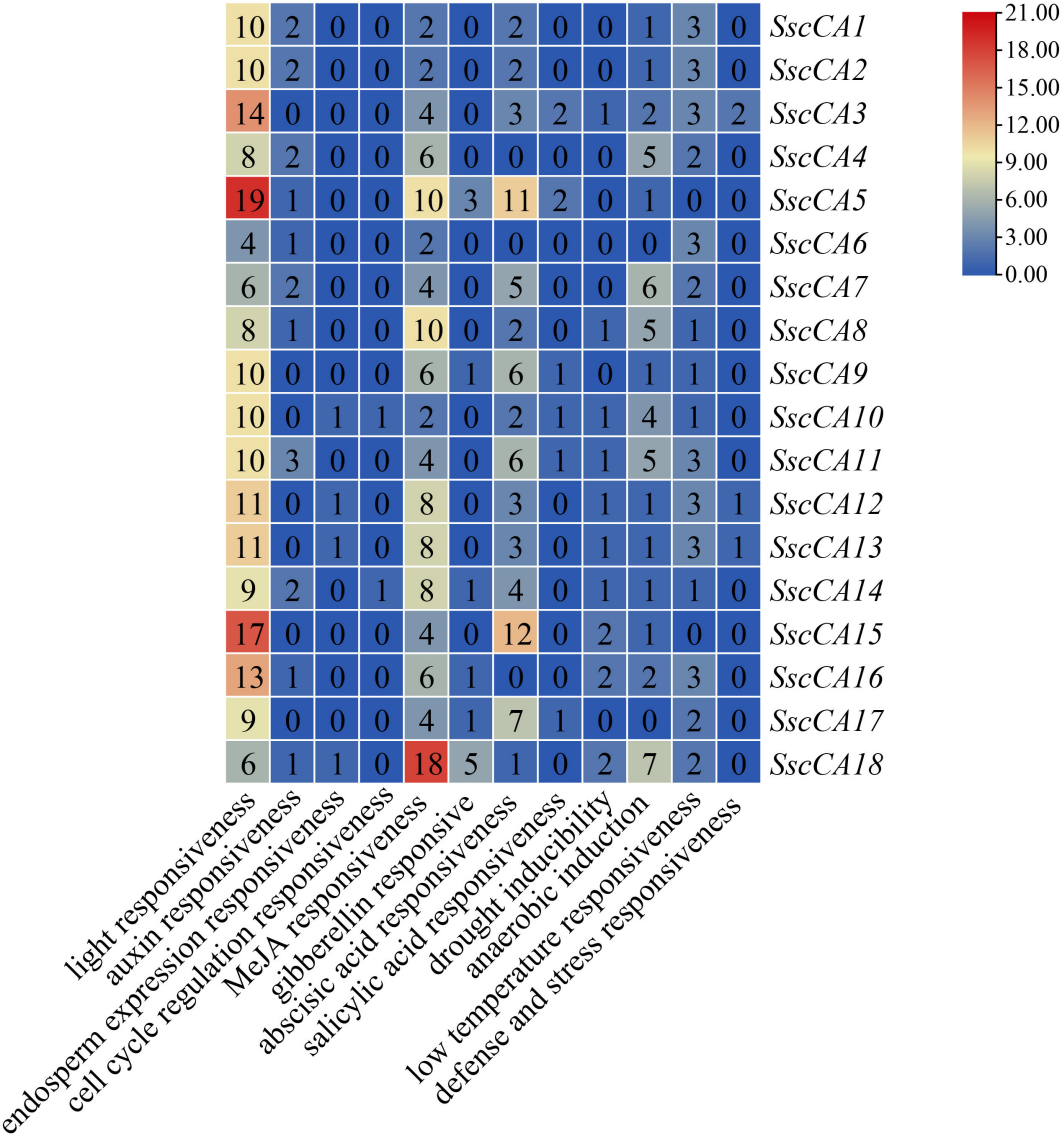
Number of distinct cis-acting elements in the promoter regions of *CA* genes.

To investigate the regulatory roles of CAs, cis-acting elements in the promoter regions were predicted and analyzed (**Figures 5**, **6**). According to functional annotations, the cis-acting elements of *CA* genes were broadly classified into four categories, including light-responsive elements (185), hormone-responsive elements (197), stress-responsive elements (97), and development-related elements (24). While diverse cis-acting regulatory elements were identified across *CA* genes, light-responsive cis-elements were universally present in all genes. Among the hormone-responsive elements, methyl jasmonate responsive elements were the most abundant (108 in total) and were detected in all *CA* genes. The next most frequent were abscisic acid responsive elements, with 69 in total, which were present in all *CA* genes except *SscCA4*, *SscCA6*, and *SscCA16*. The 97 stress-responsive elements mainly comprised low temperature responsive cis-acting elements, hypoxia and drought related elements, along with a small number of defense and stress related cis-acting elements.

### 3.6 RT-qPCR and carbonic anhydrase activity measurement

**Figure 7:**
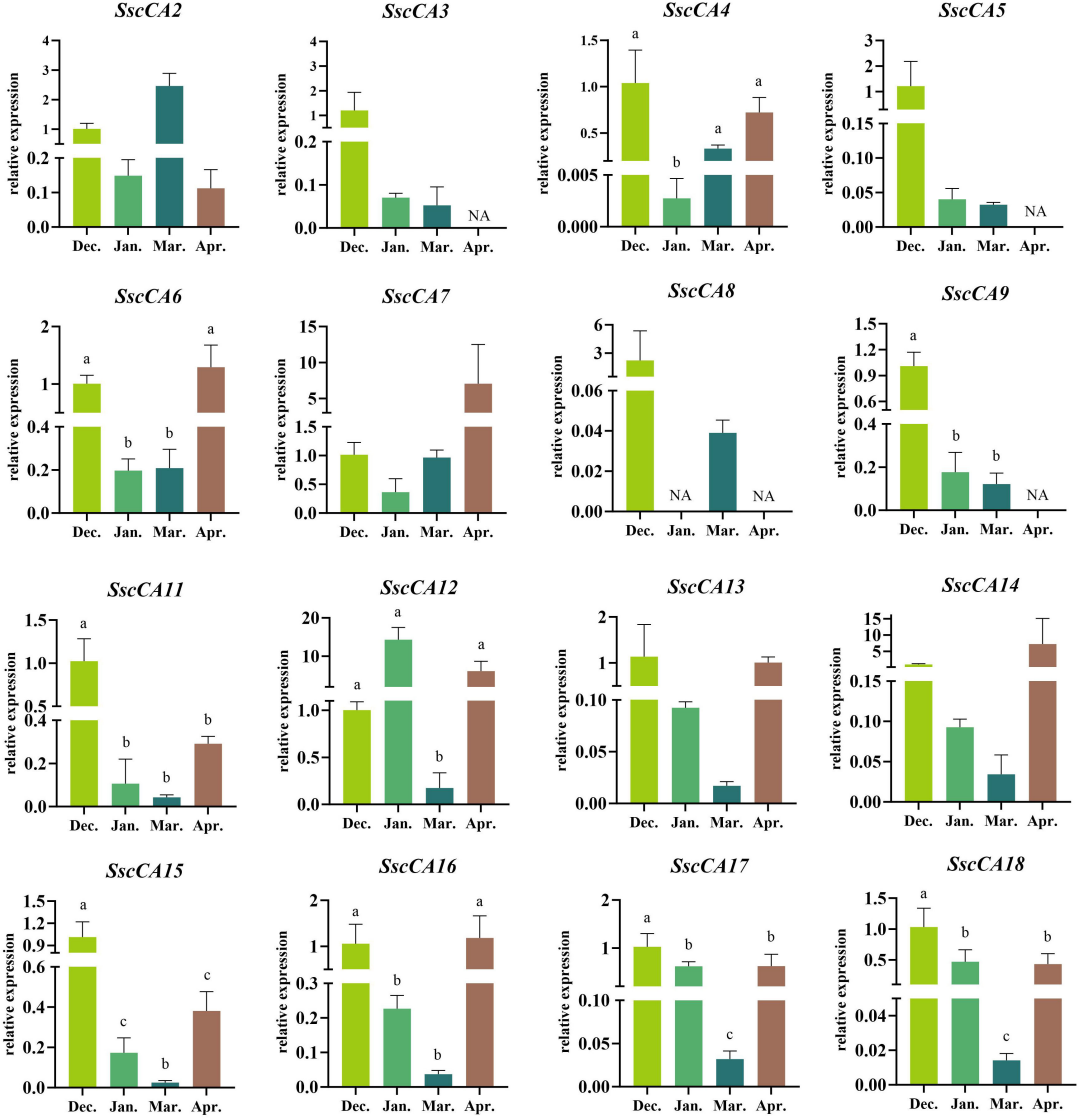
Expression pattern of CAs. “NA” stands for no data. The lowercase letters above the bars indicate statistical significance. Dec. stands for December; Jan. stands for January; Mar. stands for March; Apr. stands for April.

**Figure 8:**
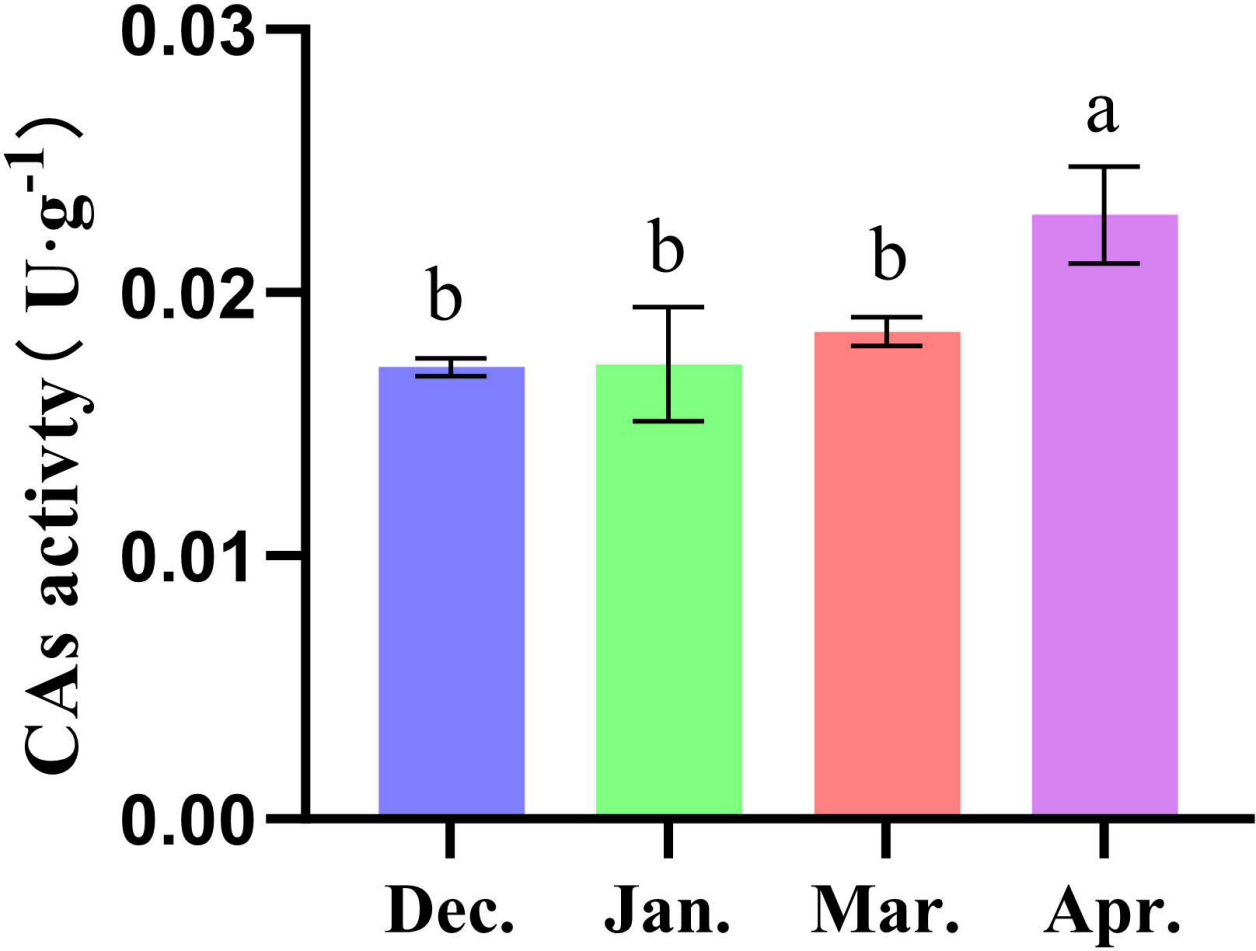
Determination of CAs activity. The lowercase letters above the bars indicate statistical significance. Dec. stands for December; Jan. stands for January; Mar. stands for March; Apr. stands for April.

The RT-qPCR was used to analyze the expression levels of *CA* genes at different developmental stages (**Figure 7**). Among all tested samples, *SscCA1* and *SscCA10* were not expressed at any stage. *SscCA3*, *SscCA5*, and *SscCA9* were undetectable in April, while *SscCA8* showed no expression in January and April. According to the results shown in **Figure 7**, the expression patterns of *CA* genes can be classified into two distinct categories. The first group, including *SscCA2*, *SscCA4*, *SscCA6*, *SscCA7*, *SscCA11*, *SscCA12*, *SscCA13*, *SscCA14*, *SscCA15*, *SscCA16*, *SscCA17*, and *SscCA18*, displayed an initial decrease followed by an increase in expression. The second group, consisting of *SscCA3*, *SscCA5*, *SscCA8*, and *SscCA9*, exhibited a continuous decline in expression. Notably, the expression levels for most genes in December were significantly different from those in January and March. Only *SscCA12* exhibited an insignificant difference in expression between December and January. However, several genes, including *SscCA15*, *SscCA16* and *SscCA18*, presented a gradual hierarchical difference in expression from January to March and April. On the other hand, measurement of the total CA activity (**Figure 8**) revealed that CA activity in April of the following year was significantly higher than that at all other sampling time points.

## 4 Discussion

CAs constitute a ubiquitous gene family in plants, playing indispensable roles in photosynthesis, respiration, plant growth and development, as well as responses to abiotic stresses (Fabre et al., 2007; De Simone and Supuran, 2012). To date, the *CA* gene family has been identified in various algae, including *C. reinhardtii*, *P. tricornutum*, and *G. chorda*, among others (Price et al., 1998; Moroney et al., 2011; Tachibana et al., 2011; Aspatwar et al., 2018; Lee et al., 2018). However, relevant studies remain limited in brown algae (Bi et al., 2021), and there has been no characterization of the *CA* gene family reported in *S. sculpera* to date. To address this gap, we employed a high-quality chromosome-level genome assembly of *S. sculpera* generated by integrating of Illumina short-reads, PacBio HiFi, and high-throughput chromosome conformation capture (Hi-C) data (Guo et al., 2025). This effectively mitigated inaccuracies in gene family identification due to poor genome quality (Yang et al., 2025), providing a solid foundation for the subsequent bioinformatics analysis.

In this study, a phylogenetic tree was constructed based on the 18 identified CAs together with their homologs from other species, revealing three distinct subfamilies of CAs (**Figure 1**), with *S. sculpera* containing more *CA* gene members than representative algal species. This might be attributed to its highly duplicated genome, in which repetitive sequences account for 60.26% of the total genome (Guo et al., 2025). Physicochemical properties of the CA family were consistent with their functional characteristics in catalyzing CO_2_-related reactions (**Table 2**). Among them, the lipophilic and hydrophobic proteins *SscCA16* and *SscCA18* were speculated to be involved in lipid metabolism (Hoang and Chapman, 2002). Gene structure analysis revealed that the arrangement and number of conserved motifs were highly similar within the same CA subfamily but differed substantially among distinct subfamilies (**Figure 2**). These results suggested that different subfamilies maintained relative independence during evolution, aligning with findings on CAs in other species (Hopkinson et al., 2016). Subcellular localization predictions indicated that CAs were mainly distributed in chloroplasts and mitochondria (**Table 2**), implying their close association with carbon assimilation and oxidative phosphorylation (Wang et al., 2024b). Furthermore, the β-CA subfamily was exclusively localized in chloroplasts, suggesting that this subfamily is closely involved in photosynthesis (Sharma et al., 2023). The CAs were also detected in the nucleus and cytoplasm, consistent with findings in other species (Moroney et al., 2011; Hopkinson et al., 2016; Bi et al., 2019; Feng et al., 2020). The uneven distribution of CAs in cells might enhance the ability of cells to respond to internal and external environmental changes (Ignatova et al., 2019).

Compared with other algae, *S. sculpera* possessed a larger number of *CA* genes. Analysis of the duplication patterns of the *CA* gene family identified 13 genes resulting from tandem duplications, suggesting that the expansion of this family is primarily driven by such events (**Figure 3**). Additionally, an investigation into the geological period of *S. sculpera*’s divergence revealed that during the Neogene, when this species arose (unpublished data), the atmospheric carbon dioxide concentrations were relatively low level and continued to decline (Hönisch et al., 2023). This environmental condition might have contributed to the increase of *CA* gene numbers in *S. sculpera*, aiding its adaptation and survival. Selective pressure analysis of the CA family showed that the Ka and Ks values of *SscCA1*/*SscCA2* and *SscCA12*/*SscCA13* were both 0 (**Table 3**). These results implied that these gene pairs originated from recent tandem duplication events, with no base mutations accumulated (Hurst, 2002). The Ka/Ks values of the other tandemly duplicated gene pairs were less than 1, indicating that CAs in *S. sculpera* have undergone purifying selection during evolution, implying that their functions are under strong evolutionary constraints (Hurst, 2002; Wang et al., 2024a).

Gene duplication often results in changes to gene expression patterns, with the original functions of these genes either being retained or diverging functionally (Kong et al., 2007). In this study, all tandemly duplicated gene pairs with Ka/Ks < 1 showed either the acquisition of new responsive elements or the loss of existing ones, which may be primarily related to responses to phytohormones and environmental stresses. Moreover, most tandemly duplicated genes exhibited distinct expression levels at the same developmental stage (**Figure 7**). These findings suggested that tandem duplication has led to functional divergence among these genes, enabling the evolution of novel response mechanisms or even new functions to facilitate adaptation to complex and fluctuating environments (Birchler and Yang, 2022). Furthermore, from the perspective of collinearity relationships between *S. sculpera* and other species (**Figure 4**), the *CA* genes in *S. sculpera* appeared to have followed distinct evolutionary paths compared with those in other algae and plants. Therefore, investigating their homologous relationships is critical for gaining deeper insights into the evolutionary history of this gene family in *S. sculpera*.

The expression patterns of several CAs varied significantly across different development stages (**Figure 7**), suggesting that development strongly influences transcriptional regulation. Notably, most chloroplast-localized *CA* genes showed an initial decline in expression in the early stage in January and a subsequent increase in the later stage in April. In contrast, most mitochondrial-localized *CA* genes displayed a continuous decrease in expression throughout all developmental stages (**Table 2** and **Figure 7**). These patterns might reflect a developmental metabolic reprogramming that would benefit carbon assimilation in chloroplasts, while suppressing mitochondrial respiratory metabolism. Such changes could promote carbohydrate accumulation and thallus growth during later stages of seaweed development (Ye et al., 2013; Zhang et al., 2020b; Bi et al., 2021). Additionally, analysis of the cis-acting elements revealed that environmental factors might also play crucial roles in regulating CA expressions (**Figures 5**–**7**). However, although cis-acting element prediction supported the universal regulation of CA expression by light signals (**Figures 5** and **6**), their expression levels did not correlated with the elevation of light intensity and light period (**Supplementary Material**), implying a complex regulatory network involving multiple light responsive cis-acting elements or modification by other factors such as phytohormones (Rudenko et al., 2017). Meanwhile, although “low temperature responsiveness” cis-acting elements were not the most abundant, the expression levels of most *CA* genes decreased synchronously from December to January as the temperature dropped (**Figures 5**–**7**), indicating that low temperature plays a critical role in suppressing CA expression during these periods (Li et al., 2026). Collectively, these findings suggested that the CA expression patterns are determined by the combined effects of developmental stages and environmental factors.

For CA enzyme activity assays (**Figure 8**), the significantly enhanced catalytic activity observed in April suggested an increased capacity for inorganic carbon supply to support carbon assimilation during later developmental stages, paralleling the trends indicated by gene expression data. Notably, the total enzymatic activity of CA did not fully correspond to the gene expression level in January and March. This discrepancy might imply the complexity of CA regulation and indicated that post-transcriptional, or post-translational modifications might modulate CA enzyme activity (Zhang et al., 2020a).

## 5 Conclusions

In this study, we performed a comprehensive analysis of the *CA* gene family in *S. sculpera*, revealing 18 CA members predominantly located on chromosomes LG11 and LG15. These genes were categorized into three subfamilies, including α-CA, β-CA and γ-CA. These *CA* genes have significantly expanded through tandem duplications and have also undergone purifying selection under natural environments. Synteny analysis revealed that *CA* genes were most closely related to those in *S. japonica*. Furthermore, the expression profiles and measured enzyme activities of CAs across different developmental stages suggested that the regulatory mechanism governing the *CA* gene family is complex in *S. sculpera*. Overall, these results provided new insights into the biological functions of CAs. They also lay a solid foundation for future studies exploring the roles of CAs in the growth, development and evolution of *S. sculpera*.

## Data Availability

The original contributions presented in the study are included in the article/**Supplementary Material**, further inquiries can be directed to the corresponding author/s.
